# Mucinous Cystadenoma: A Rare Hepatic Tumor in a Child

**DOI:** 10.3389/fped.2017.00215

**Published:** 2017-10-09

**Authors:** Danielle A. Ferraguti, Molly McGetrick, Ivan Zendejas, David Hernandez-Gonzalo, Regino Gonzalez-Peralta

**Affiliations:** ^1^Division of Gastroenterology, Hepatology and Nutrition, Department of Pediatrics, University of Florida, Gainesville, FL, United States; ^2^General Education, Department of Pediatrics, University of Florida, Gainesville, FL, United States; ^3^Division of Hepatobiliary Surgery, Intermountain Medical Center, Canyon Surgical Associates, Murray, UT, United States; ^4^Division of Surgery, Intermountain Medical Center, Canyon Surgical Associates, Murray, UT, United States; ^5^Department of Pathology, University of Florida, Gainesville, FL, United States

**Keywords:** liver mass, children, pediatrics, mucinous cystadenoma, liver tumors

## Abstract

Mucinous cystadenomas (MCAs) of the liver (also called hepatic biliary cystadenomas) are rare tumors that comprise about 5% of cystic masses of the liver in adults. These slow-growing lesions most commonly occur in middle-aged individuals, with a female sex predominance. Herein, we present a MCA in a 6-year-old male, one of only very few such cases described in the pediatric literature to date. Although MCAs are generally considered benign lesions, malignant transformation rarely occurs. The recurrence rate is high when partial cyst excision is performed. Therefore, complete surgical cyst resection with clinical follow-up, including imaging, is warranted.

## Introduction

Mucinous cystadenomas (MCAs) of the liver are rare intrahepatic lesions seldom discovered in childhood. These lesions are typically found incidentally, often during evaluation of non-specific abdominal complaints. Like other more widely described hepatic masses, MCAs can cause symptoms such as abdominal pain, nausea, vomiting, and jaundice from mass effect. These “great pretenders” are easily mistaken for abscesses, cysts, or even malignancy, which prompts diagnostic resections. Characteristically, MCAs are considered benign, although secondary malignant transformation has occurred in as many as 20% of affected adults (1). Definitive treatment is complete surgical resection with procedures that aim to prevent significant distortion of liver architecture and alterations in function, followed by close clinical surveillance.

Herein, we report a case of a 6-year-old healthy male diagnosed with MCA after a liver mass was incidentally discovered during an echocardiogram. Through this report, we hope to educate gastroenterologists and surgeons about a rare mass that they may encounter more frequently in practice as abdominal imaging modalities improve. We will discuss the role of imaging and histology in the diagnosis of MCAs, definitive management, and propose a standard for routine surveillance in affected patients.

## Case Presentation

A 6-year-old previously healthy male presented to the Pediatric Gastroenterology Clinic for evaluation of a liver mass that was incidentally seen on an echocardiogram while assessing a heart murmur. At the time of the discovery of the liver lesion, the patient was asymptomatic, without abdominal pain, anorexia, weight loss, vomiting, jaundice, or other liver-related complaints. Vaccinations, including hepatitis B, were up to date.

Findings on physical examination were normal. Results of serum blood chemistry including markers of liver synthetic function (INR, albumin), transaminases, bilirubin, alpha-fetoprotein (AFP), cancer antigen (CA19-9), and carcinogen embryonic antigen (CEA) were normal. HAV, HBV, and HCV serologies were also negative. An abdominal ultrasound showed a cystic lesion with irregular borders in the left hepatic lobe. The hepatic mass was characterized by computerized tomography (CT) as a circumscribed, complex, multi-lobulated, and septated cystic lesion measuring 5.5 cm × 4.2 cm × 5.2 cm.

**Figure 1:**
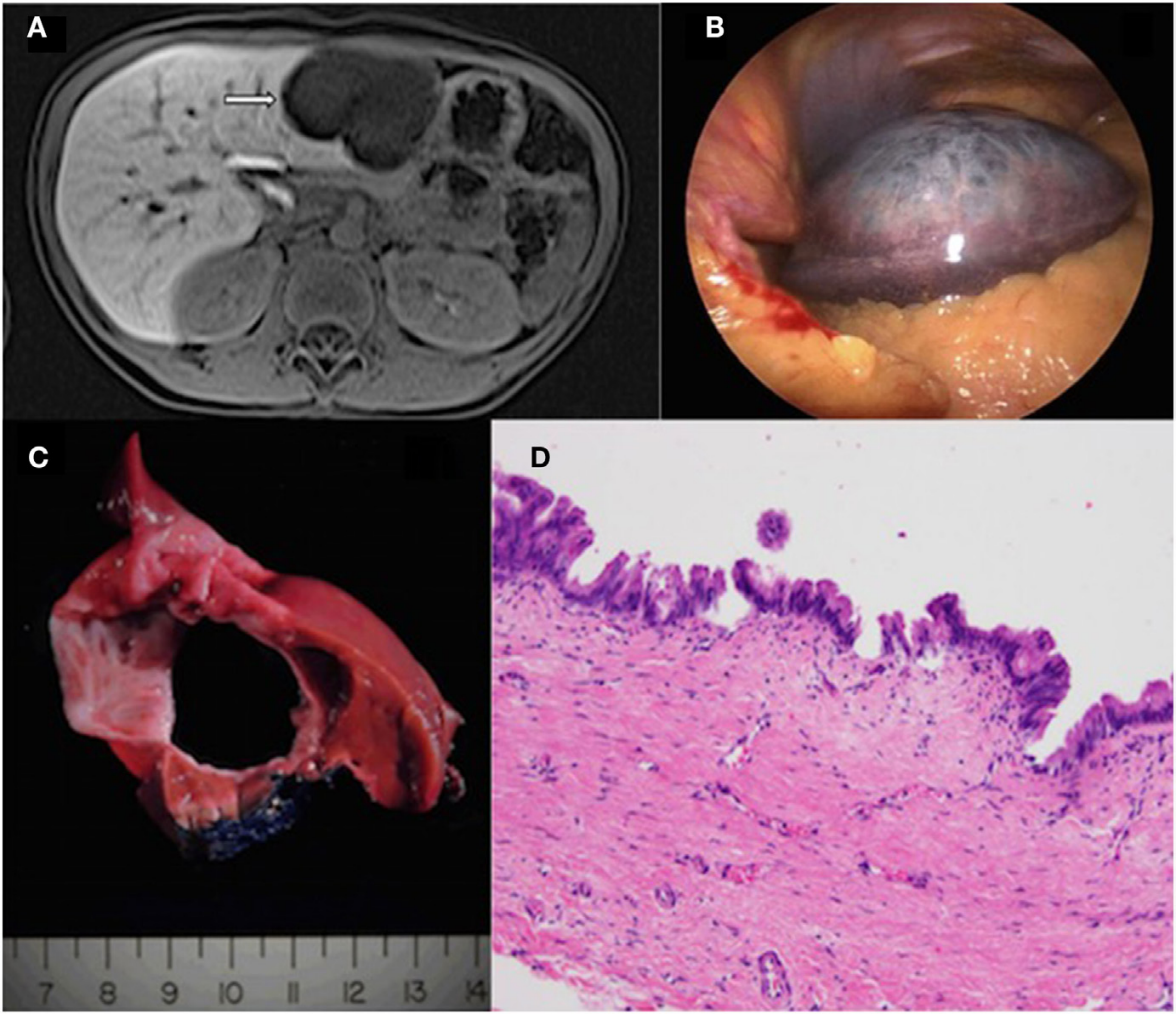
**(A)** Magnetic resonance imaging of the liver (with Eovist contrast) showing a cystic lesion in the left hepatic lobe measuring 6.0 cm × 6.1 cm × 3.8 cm. There is thin enhancement of septations seen within the lesion. **(B)** Gross pathology showing a large, well-circumscribed mass with surrounding liver parenchyma. **(C)** Serial sectioning of the mass revealed a cyst-like appearance with clear serous fluid filling the central portion. **(D)** The resected cyst is lined by mucin-type epithelium ranging from low cuboidal to stratified-columnar with frequent papillary tufting. No significant nuclear pleomorphism or loss of nuclear polarity is noted. The histologic findings are best classified as mucinous cystic neoplasm with low-intermediate grade dysplasia (original magnification 20×, hematoxylin and eosin).

Abdominal magnetic resonance imaging (MRI) done 7 months later showed the same complex cystic lesion in the left hepatic lobe without interval change in size. Given the absence of symptoms and benign laboratory and imaging results, we pursued a conservative approach with clinical, biochemical, and radiological follow-up every 6 months. Results of the patient’s laboratory tests remained normal, but the lesion showed slight growth on ultrasound over the following 2.5 years. A repeat abdominal MRI (with Eovist contrast) showed the complex multi-cystic lesion had increased slightly in size to 6.1 cm × 4.1 cm × 6.4 cm (Figure 1A).

Due to the enlargement and complex nature of the lesion, the patient was referred to our hepatobiliary surgeons for resection. Exploratory laparotomy showed normal exterior hepatic tissue but a mass was palpable beneath the left hepatic lobe. A hepatic laparoscopic ultrasound showed that the tumor supplanted the parenchyma of the lateral segment of the liver. As a result, a left lateral hepatectomy was performed with complete excision of the mass. The patient tolerated the procedure well and was discharged home on postoperative day 3.

Gross histopathological assessment revealed a well-circumscribed 7.3 cm × 5.8 cm mass (Figure 1B). Serial sectioning revealed clear serous fluid within the cystic lesion as well as a ragged white appearance of the cyst lining (Figure 1C). The walls lacked papillary excrescences or solid mural nodules. Microscopic evaluation revealed a cuboidal to columnar variably mucin-producing epithelium with low-grade dysplasia (no significant atypia) with frequent ondulations of the cyst lining. Adjacent to the cyst lining, there was a fibrotic stroma. There was no distortion of the surrounding hepatic architecture or evidence of hepatic fibrosis (Figure 1D).

Our patient returned to clinic every 6 months for routine physical exam, laboratory work, and ultrasound of the liver. Seventeen months after the tumor resection, the patient remained asymptomatic. MRI of the abdomen was performed at 19 months, which showed only expected postsurgical changes and no recurrence of the mass. His tumor markers, including AFP, CEA, and CA 19-9 remained within normal limits, as did markers of synthetic function. He continues to have regular follow-up at 6-month intervals.

## Discussion

Hepatic MCAs are benign tumors that are often discovered incidentally on imaging. These hepatic tumors make up approximately 5% of cystic hepatic lesions of the liver in the adult population (1–8). The precise incidence in children is unknown, but it is a rare disorder in this group, with few cases reported in the literature. The paucity of reports can be at least partially explained by the fact that MCAs are slow growing tumors that present with mild and non-specific symptoms that may be unrelated to the liver, as in our patient. However, the increased use of abdominal imaging in clinical practice has led to a rise in case identification (7, 9). The pathogenesis of hepatic mucinous cystadenoma is unknown. Early reports suggested a hormonal influence on the development and growth of these hepatic lesions that were primarily described in middle-aged women. However, the presence of MCAs in children as young as 2 years old argues against hormonal effect and invokes a connection with congenital anomalies or aberrant growth in the progenitor cells of the hepatic and biliary systems (1, 2, 10). In this context, MCAs are associated with other anomalies in the gastrointestinal, biliary, and respiratory tracts, as well as genitourinary systems (2, 10).

Mucinous cystadenomas have an insidious nature and are often asymptomatic or present with vague symptoms. Large tumors can give rise to symptoms related to mass effect, including abdominal pain and clinical signs of biliary obstruction. The most common findings at diagnosis are abdominal pain (60–90%), palpable abdominal mass (up to 53%), nausea, vomiting and rarely, jaundice (1, 4–6, 10, 11). In cases that present with severe jaundice, prolapse into the bile ducts may be noted. Because of the rapid onset of symptoms, masses with this growth pattern seem to be diagnosed at earlier ages (12). Results of liver tests, CA19-9 and CEA are typically normal (3, 10).

On imaging, lesions may be unilocular or multilocular and resemble abscesses, echinococcal cysts, teratomas, hematomas, necrotic tumors, and polycystic disease (1, 2, 10, 11, 13). Intrahepatic lesions predominate over biliary lesions, and the majority of masses are found in the right lobe of the liver (3, 4). Abdominal ultrasound is usually the first imaging modality employed to evaluate these lesions and show anechoic lesions with multiloculated areas and septations (in greater than 80%) with a “cyst within a cyst” appearance (in more than 50%) (7). Less commonly, irregular cyst walls, papillary projections, and calcifications may be present. On CT, isodense lesions with nodular areas of enhancement are typical. Although the appearance of solid components, irregular walls, or mural septations is more suggestive of malignancy, none of these findings are reliable to exclude liver cancer (1, 6, 11).

Histologically, MCAs are usually lined with cuboidal or non-ciliated columnar cells, with basal nuclei and mucin-containing cytoplasm (1, 4, 13). The epithelial cells lining MCAs contain variable dysplasia; therefore, they are categorized into low- and high-grade dysplasia. Moderate dysplasia is a term that is no longer recommended. It has been hypothesized that these tumors arise from primitive ectopic rests of bile ducts, although 50% of specimens contain endocrine cells, suggesting a glandular origin (1). Historically, MCAs were notorious for containing ovarian-like stroma that is vascular and dense with spindle-shaped cell on histopathology. Not surprisingly, case reports of female patients predominated (1, 11). However, emerging literature describes a second variety of tumor without a mesenchymal layer, but rather a hyaline, fibroid, or myxoid stroma (2, 14). Neither the case we present, nor 10 of the 11 previously described cases of MCA in children contained ovarian-like stroma. In this second group of tumors, there is no significant gender predominance but notably a worse prognosis, further supporting the rationale for surgical resection in management (6, 15).

Because it is difficult to diagnose MCAs by imaging, the gold standard for treating these lesions is complete surgical resection with confirmed clear margins (1, 5, 9). Operative management includes total resection or procedures that attempt to preserve liver architecture. Liver-preserving procedures include marsupialization, fenestration of cystic components, and partial tumor removal. According to a small adult study, patients who underwent liver-sparing procedures had greater than a 60% rate of cyst recurrence (7). Additionally, the rate of malignant transformation of tumor tissue has been reported as high as 20% in the adult literature, although this has not been described in pediatric patients (1). Therefore, complete resections should be considered provided that the remaining hepatic tissue is sufficient in quantity and is not compromised by infection, cirrhosis, or any other illness that may adversely affect postsurgical hepatic function (1).

Percutaneous biopsy and analysis of cystic contents have been proposed as a less invasive diagnostic option (1, 7, 8). For example, Koffron et al. described an algorithm for diagnosis based on concentration of tumor markers in cystic fluid. Elevated levels of CA 19-9 and CEA were noted in all 22 patients with MCA in this study (8). Additionally, it has also been shown that CA 19-9 and CEA can be elevated to a variable extent in simple hepatic cysts, which is likely due to the fact that these markers are normally expressed in the biliary epithelial cells and bile ducts (16). At this time, the utility of percutaneous sampling of cystic fluid is limited in that the specificity of the currently employed markers is relatively low and there exists a theoretical, yet, highly unlikely risk of intra-abdominal seeding of potentially malignant cells. However, tumor-associated glycoprotein-72 has been proposed as a more specific marker for mucinous hepatic lesions when measured in cystic fluid prior to surgical intervention. In contrast to CA 19-9 and CEA, this protein is not expressed by normal biliary cells, either at baseline or during times of inflammation (16). Although promising, more investigations need to be performed to determine if this marker can be used in children to delineate simple hepatic cysts from MCAs requiring definitive operative resection.

In summary, given the lack of consistently reliable non-invasive diagnostic methods and high tumor recurrence rate with partial resection, complete surgical excision is considered the gold standard. Even with complete removal, the recurrence rate is 5–10% in adults (1). Although there are no standard guidelines, it seems reasonable to propose close follow-up and surveillance imaging every 3–6 months for at least 5 years after MCA resection.

## Ethics Statement

Written informed consent for the presentation and publication of this case was obtained from the patient’s legal guardian.

## Author Contributions

DF and MM drafted the manuscript. IZ edited the manuscript, and obtained and interpreted the gross pathology images. DH-G edited the manuscript and obtained and interpreted the microscopic pathology images. RG-P critically reviewed the manuscript and is the author guarantor.

## Conflict of Interest Statement

The authors declare that the research was conducted in the absence of any commercial or financial relationships that could be construed as a potential conflict of interest. The reviewer NK and handling editor declared their shared affiliation.
